# Trends in disparities in healthcare utilisation between and within health insurances in China between 2008 and 2018: a repeated cross-sectional study

**DOI:** 10.1186/s12939-022-01633-4

**Published:** 2022-02-24

**Authors:** Xiaoling Yan, Yuanli Liu, Min Cai, Qinqin Liu, Xueqin Xie, Keqin Rao

**Affiliations:** 1 School of Health Policy and Management, Chinese Academy of Medical Sciences and Peking Union Medical College, Beijing, 100730 China; 2 Institute of Medical Information, Chinese Academy of Medical Sciences and Peking Union Medical College, Beijing, 100020 China; 3 Center for Health Statistics and Information, National Health Commission, Beijing, 100044 China; 4 China Health Economics Association, Beijing, 100191 China

**Keywords:** Healthcare utilisation, Utilisation disparity, Social health insurance, Trends, Income-based inequity

## Abstract

**Background:**

Fragmentation in China’s social health insurance schemes and income gap have been recognised as important factors for the inequitable use of healthcare. This study assessed trends in disparities in healthcare utilisation between and within health insurances in China between 2008 and 2018.

**Methods:**

We used data from the 2008, 2013, and 2018 China National Health Services Survey. Outpatient visit, inpatient admission and foregone inpatient care were chosen to measure healthcare utilisation and underutilisation by health insurances. Absolute differences and rate ratios were generated to examine disparities between and within health insurances, and changes in disparities were analysed descriptively. Pearson χ2 tests were used to test for statistical significance of differences.

**Results:**

The outpatient visit rate for respondents covered by the urban resident-based basic medical insurance scheme (URBMI) more than doubled between 2008 and 2018, increasing from 10.5% (9.7-11.2) to 23.5% (23.1-23.8). Inpatient admission rates for respondents covered by URBMI and the new rural cooperative medical scheme (NRCMS) more than doubled between 2008 and 2018, increasing by 7.2 (*p* < 0.0001) and 7.4 (*p* < 0.0001) percentage points, respectively. Gaps in outpatient visits and inpatient admissions narrowed across the urban employee-based basic medical insurance scheme (UEBMI), URBMI, and NRCMS through 2008 to 2018, and by 2018 the gaps were small. The rate ratios of foregone inpatient care between NRCMS and UEBMI fell from 0.9 (*p* > 0.1) in 2008 to 0.8 (*p* < 0.0001) in 2018. Faster increases in outpatient and inpatient utilisation and greater reductions in foregone inpatient care were observed in poor groups than in wealthy groups within URBMI and NRCMS. However, the poor groups within UEBMI, URBMI, and NRCMS were always more likely to forego inpatient care in comparison with their wealthy counterparts.

**Conclusions:**

Remarkable increases in healthcare utilisation of URBMI and NRCMS, especially among the poorest groups, were accompanied by improvements in inequality in healthcare utilisation across UEBMI, URBMI, and NRCMS, and in income-based inequality in healthcare utilisation within URBMI and NRCMS. However, the poor groups were always more likely to forego admission to hospital, as recommended by doctors. We suggest further focus on the foregoing admission care of the poor groups.

**Supplementary Information:**

The online version contains supplementary material available at 10.1186/s12939-022-01633-4.

## Background

Inequitable access to quality healthcare services has important implications for health inequalities. China’s recent round of healthcare system reforms launched in 2009 pledge to provide accessible and affordable basic healthcare services for all citizens by 2020 [1]. Among its ambitious healthcare reform plans, one of the priorities was government-led health insurance reforms to achieve universal health coverage [1–3]. China launched three different social health insurance schemes successively, namely, the urban employee-based basic medical insurance scheme (UEBMI; introduced in 1997), new rural cooperative medical scheme (NRCMS; introduced in 2003), and urban resident-based basic medical insurance scheme (URBMI; introduced in 2007), with different target populations [3]. Household registration and employment status determine residents’ insurance scheme. In 2008, 87.9% of the Chinese population was covered by UEBMI, URBMI and NRCMS [4]. The recent round of healthcare system reforms rapidly expanded the coverage of UEBMI, URBMI and NRCMS through strong political commitment and extensive financial subsidies from the government. By the end of 2011, 96.9% of the Chinese population was covered by UEBMI, URBMI and NRCMS, and the coverage rate has remained above 95% ever since [5]. However, UEBMI, URBMI and NRCMS have separate fund pools, different service coverage, and financial protection. UEBMI has more generous benefits than URBMI and NRCMS [1, 6]. Integrating URBMI and NRCMS to Urban-Rural Resident Basic Medical Insurance (URRBMI) was piloted in some provinces and municipal cities, and was announced by the central government in 2016 with the gradual implementation of consolidation nationwide [3, 7]. However, statistics from the National Healthcare Security Administration show that more than one-fifth of provinces (seven of the 31 provinces) still implemented NRCMS in 2018.

Fragmentation in China’s social health insurance schemes and income gap have been recognised as important factors for the inequitable use of healthcare for people covered under different health insurance schemes [3, 6–8]. Previous studies have investigated insurance-related and income-related inequity in healthcare utilisation [7–17]. Consensus is yet to be reached on the inequitable use of healthcare among health insurance schemes and income groups. Some studies claimed that there were gaps in healthcare utilisation among different health insurances [9–12]. Other studies showed that outpatient service utilisation was almost identical among UEBMI, URBMI and NRCMS [8, 13], and there were no significant differences in outpatient and inpatient services utilisation between URRBMI and NRCMS [7]. Some studies have found that NRCMS and URBMI have improved income-based inequalities in healthcare utilisation [14–17]. However, most of them examined insurance-related and income-related differences in healthcare utilisation at a cross-sectional level.

Few studies have focused on the trends in disparities in healthcare utilisation among health insurances in China, and changes in income-based disparities within health insurances have not been captured [6]. Existing studies have examined trends in residents’ healthcare utilisation and paid attention to changes in urban-rural and income-based disparities in healthcare utilisation [1, 18, 19] or changes in urban-rural differences in healthcare utilisation among insured older adults [20]. To date, little is known about the trends in disparities in healthcare utilisation between and within health insurances.

In this study, we use data from the three latest waves of the China National Health Services Survey (CNHSS) to examine changes in disparities in healthcare utilisation between health insurances and changes in income-based disparities within UEBMI, URBMI, and NRCMS between 2008 and 2018. Our findings provide empirical support for further reforms of China’s health insurance system. The results also identify lessons for the development of health insurance systems in other countries.

## Methods

### Data source

The data employed in this study was derived from three latest waves (2008, 2013, and 2018) of CNHSS. The Chinese National Health Commission (originally called the Ministry of Health) has conducted a national household health service survey every 5 years since 1993, and collected comprehensive information about demographic and socioeconomic characteristics, self-reported health status, healthcare utilisation, and health behavioural factors. In this study, we primarily used information on healthcare utilisation. A multistage cluster sampling method was used to select households for the interviews. Stratified by province, 94, 156, and 156 counties in 31 provinces, autonomous regions and municipalities in mainland China were randomly selected from 2859 counties in 2008, 2013, and 2018, respectively. In every county, five streets or towns were randomly chosen, and two residential committees or villages were selected in every selected street or town, from which 60 households were randomly selected from each residential committee or village. The 2008 survey was conducted in June and July, and the 2013 and 2018 surveys were conducted in September. Within every household, all members aged 15 years and older completed an interview utilizing a structured questionnaire, and questions about children younger than 15 years were answered by adult family members. The response rate at the household level was above 95% for each survey. Local medical workers were recruited and trained to conduct face-to-face interviews and were supervised by doctors from township hospitals or higher-level health institutions to ensure data quality. Of these, 5% of the households were revisited to examine consistencies of data, and the consistencies were higher than 95% in each survey [21, 22]. Existing studies have reported high reliability, validity, and other aspects of data quality in CNHSS [21–25].

### Study population and measures

According to our research purpose, data were disaggregated by health insurance coverage status. The definitions of health insurance coverage statuses of respondents were provided in the online supplementary material [see Appendix A in Additional file 1]. Results report data for UEBMI, URBMI, NRCMS, and uninsured residents (respondents without any kind of health insurance). Data were also disaggregated by income levels within the UEBMI, URBMI and NRCMS groups, which have a sufficient sample size to detect changes by income groups. Using the method of five levels of income classification, all respondents in each county were equally divided into five income groups based on the distribution of their self-reported household annual income per capita. Then, respondents in the same income group from all counties were aggregated to form five income groups (low-income, lower- middle-income, middle-income, upper-middle-income, and high-income groups) within UEBMI, URBMI, and NRCMS groups. The same method of five levels of income classification was applied across all three social health insurance schemes in every wave of the survey.

We used outpatient visits, inpatient admissions and foregone inpatient care to measure healthcare utilisation. Table A1 in Additional file 1 provides the definitions of these three indicators, which were measured in the same way in every wave of the survey. We measured trends in healthcare utilisation by health insurances, estimating absolute differences between each consecutive survey year, and between 2008 and 2018. We examined the changes in disparities in healthcare utilisation between and within health insurances. To study the change in disparities across health insurances, the set of six comparators were UEBMI and uninsured residents, URBMI and uninsured residents, NRCMS and uninsured residents, UEBMI and URBMI, UEBMI and NRCMS, and URBMI and NRCMS. To study the change in income-based disparities within the health insurances, the comparator was income quintile 1 (poor) and quintile 5 (least poor) for UEBMI, URBMI, and NRCMS. Ratios were generated by dividing the rates for the above three indicators of healthcare utilisation for each comparison group. The ratio indicates the disparity between the comparison groups, where ‘1’ implies no gap [21]. The disparity is considered ‘widening’ if the ratio is moving away from ‘1’ and ‘narrowing’ if the ratio approaches ‘1’, regardless of whether the initial value is greater or less than ‘1’ [21]. This study also reports changes in absolute differences among the comparison groups.

### Statistical analysis

Statistical analyses were performed using the SAS software 9.4. We used Pearson χ2 tests to test for the statistical significance of trends between each consecutive survey year and between 2008 and 2018. Pearson χ2 tests were also used to test for statistical significance of disparities between health insurances and between quintile 1 (poor) and quintile 5 (least poor) within the UEBMI, URBMI, and NRCMS in 2008, 2013, and 2018. We reported significant values and 95% CIs for percentages.

## Results

### Characteristics of study participants

Table 1 presents the basic characteristics of the UEBMI, URBMI, NRCMS, and uninsured respondents in 2008, 2013, and 2018. For each year, the proportion of male was slightly higher than that of female for UEBMI, while the reverse was true for URBMI and the uninsured respondents; the proportion of male and female was almost identical for NRCMS. In terms of the proportion of the population aged 65 and above, UEBMI had the highest; URBMI and NRCMS were comparable; and the uninsured respondents had the lowest. The percentage of the population aged 65 years and above increased over waves for all study groups. Take NRCMS as an example, the percentage of the population aged 65 years and above increased from 9.7% in 2008 to 17.8% in 2018. The age-group compositions of UEBMI, URBMI, NRCMS, and uninsured respondents in 2008, 2013, and 2018 were shown in Fig. A1 in Additional file 2. UEBMI had the highest per capita household annual income, followed by URBMI and uninsured residents, with NRCMS having the lowest. Annual household income per capita grew annually by 6.5, 5.4, 7.5, and 7.8% for UEBMI, URBMI, NRCMS and uninsured respondents, respectively.

**Table 1:** Characteristics of the UEBMI, URBMI, NRCMS, and uninsured respondents^a^

	2008		2013		2018	
	***n***	%(95% CI)	***n***	%(95% CI)	***n***	%(95% CI)
Sample size						
UEBMI	22,520	–	57,389	–	60,176	–
URBMI	6722	–	37,269	–	62,598	–
NRCMS	121,870	–	141,513	–	125,421	–
Uninsured	21,466	–	10,502	–	6290	–
Sex(=male)						
UEBMI	11,710	52.0(51.4-52.7)	29,878	52.1(51.7-52.5)	30,951	51.4(51.0-51.8)
URBMI	3009	44.8(43.6-46.0)	17,054	45.8(45.3-46.3)	29,603	47.3(46.9-47.7)
NRCMS	60,792	49.9(49.6-50.2)	70,230	49.6(49.4-49.9)	61,839	49.3(49.0-49.6)
Uninsured	10,465	48.8(48.1-49.4)	5172	49.3(48.3-50.2)	3106	49.4(48.1-50.6)
Age (≥65 year)						
UEBMI	5275	23.4(22.9-24.0)	13,529	23.6(23.2-23.9)	14,808	24.6(24.3-25.0)
URBMI	675	10.0(9.3-10.8)	4506	12.1(11.8-12.4)	9680	15.5(15.2-15.8)
NRCMS	11,785	9.7(9.5-9.8)	17,281	12.2(12.0-12.4)	22,363	17.8(17.6-18.0)
Uninsured	1872	8.7(8.3-9.1)	648	6.2(5.7-6.7)	584	9.3(8.6-10.0)
Annual household income per capita (RMB) (mean,95%CI)^b^						
UEBMI	17,021.5(16,870.5-17,172.5)		24,651.1(24,510.7-24,791.5)		31,842.4(31,650.1-32,034.8)	
URBMI	10,829.8(10,610.7-11,048.9)		16,347.8(16,215.8-16,479.8)		18,316.7(18,175.5-18,457.9)	
NRCMS	6120.0(6089.8-6150.2)		10,820.5(10,772.4-10,868.8)		12,669.1(12,595.2-12,743.0)	
Uninsured	8211.1(8109.3-8312.9)		14,271.7(14,038.1-14,505.2)		17,344.2(16,884.9-17,803.5)	

^a^Data are presented as numbers and percentage (95% CI) unless otherwise stated

^b^Annual household income per capita were adjusted for inflation using the economy-wide consumer price index from the International Monetary Fund, and were reported in 2018 Yuan values

### Trends in healthcare utilisation by health insurances

Table 2 shows trends in healthcare utilisation by different health insurance coverage statuses between 2008 and 2018. UEBMI, URBMI, NRCMS, and uninsured respondents’ outpatient visits and inpatient admissions witnessed significant increases between 2008 and 2018, with most of the growth taking place between 2013 and 2018. The outpatient visit rate for URBMI more than doubled between 2008 and 2018, increasing from 10.5 to 23.5%, and outpatient visit rates for UEBMI, NRCMS and uninsured respondents increased by about 60%. Inpatient admission rates for URBMI and NRCMS more than doubled between 2008 and 2018, increasing by 7.2 and 7.4 percentage points, respectively. Inpatient admission rates for UEBMI and uninsured respondents increased by more than 60% between 2008 and 2018. The rates of foregone inpatient care for UEBMI, URBMI, NRCMS, and uninsured respondents experienced dramatic reductions between 2008 and 2013, while it exhibited significant increases between 2013 and 2018. Trends in age-standardised rates of outpatient visits, inpatient admissions, and foregone inpatient care for UEBMI, URBMI, NRCMS, and uninsured respondents were similar to trends in their crude rates described above (see Table A2 in Additional file 1).

**Table 2:** Trends in healthcare utilisation by health insurances, 2008–2018^1^

	2008		2013		2018		Difference (percentage points)^**2**^		
	***n***	% (95% CI)	***n***	% (95% CI)	***n***	% (95% CI)	2008-13	2013-18	2008-18
**Outpatient visit**									
UEBMI	3281	14.6 (14.1-15.0)	7681	13.4 (13.1-13.7)	13,752	22.9 (22.5-23.2)	−1.2***	9.5***	8.3***
URBMI	704	10.5 (9.7-11.2)	4625	12.4 (12.1-12.7)	14,679	23.5 (23.1-23.8)	1.9***	11.0***	13.0***
NRCMS	18,888	15.5 (15.3-15.7)	18,758	13.3 (13.1-13.4)	31,578	25.2 (25.0-25.4)	−2.2***	11.9***	9.7***
Uninsured	2310	10.8 (10.4-11.2)	931	8.9 (8.3-9.4)	1094	17.4 (16.5-18.3)	−1.9***	8.5***	6.6***
**Inpatient admission**									
UEBMI	2068	9.2 (8.9-9.6)	6453	11.2 (11.0-11.5)	8920	14.8 (14.5-15.1)	2.1***	3.6***	5.6***
URBMI	343	5.1 (4.6-5.3)	2633	7.1 (6.8-7.3)	7729	12.4 (12.1-12.6)	2.0***	5.3***	7.2***
NRCMS	8408	6.9 (6.8-7.0)	12,686	9.0 (8.8-9.1)	17,936	14.3 (14.1-14.5)	2.1***	5.3***	7.4***
Uninsured	928	4.3 (4.1-4.6)	556	5.3 (4.9-5.7)	465	7.4 (6.8-8.0)	1.0***	2.1***	3.1***
**Foregone inpatient care**									
UEBMI	628	23.3 (21.7-24.9)	1212	15.8 (15.0-16.6)	1906	17.6 (16.9-18.3)	−7.5***	1.8**	−5.7***
URBMI	144	29.6 (25.5-33.6)	592	18.4 (17.0-19.7)	2034	20.8 (20.0-21.6)	−11.2***	2.5**	−8.8***
NRCMS	2770	24.8 (24.0-25.6)	2796	18.1 (17.5-18.7)	5159	22.3 (21.8-22.9)	−6.7***	4.3***	−2.5***
Uninsured	451	32.7 (30.2-35.2)	134	19.4 (16.5-22.4)	175	27.2 (23.7-30.6)	−13.3***	7.8**	−5.5*

^1^Data are presented as total numbers and crude rates (95% CI) of healthcare utilisation unless otherwise stated

^2^***, ** and * denote *p* < 0.0001, *p* < 0.01, and *p* < 0.05

### Changes in disparities in healthcare utilisation between health insurances

Table 3 describes the changes in disparities in healthcare utilisation between health insurances between 2008 and 2018. Ratios are presented to measure the disparities in healthcare utilisation between the comparators (1 implies no difference). Table A3 in the Additional file 1 shows the absolute differences between the comparison groups. UEBMI had higher rates of outpatient visits and inpatient admissions than those of uninsured respondents in 2008, 2013, and 2018. The gaps in the rates of outpatient visits and inpatient admissions between UEBMI and uninsured respondents were stable between 2008 and 2018; UEBMI had lower rates of foregone inpatient care than uninsured respondents did in 2008, 2013, and 2018. The gaps in rates of foregone inpatient care between UEBMI and uninsured respondents increased between 2008 and 2018.

**Table 3:** Changes in disparities in healthcare utilisation between health insurances, 2008-2018

Comparison groups	Ratio between comparison groups^**1**^						Absolute change between 2008 and 2018 (percentage points)^**2**^
	2008	***p*** value^**3**^	2013	***p*** value^**3**^	2018	***p*** value^**3**^	
**UEBMI**: **Uninsured**							
Outpatient visit	1.4	< 0.0001	1.5	< 0.0001	1.3	< 0.0001	−1.7
Inpatient admission	2.1	< 0.0001	2.1	< 0.0001	2.0	< 0.0001	−2.5
Foregone inpatient care	0.7	< 0.0001	0.8	0.0135	0.6	< 0.0001	0.2
**URBMI: Uninsured**							
Outpatient visit	1.0	0.5047	1.4	< 0.0001	1.3	< 0.0001	−6.3
Inpatient admission	1.2	0.0072	1.3	< 0.0001	1.7	< 0.0001	−4.1
Foregone inpatient care	0.9	0.2017	0.9	0.5141	0.8	0.0001	3.3
**NRCMS: Uninsured**							
Outpatient visit	1.4	< 0.0001	1.5	< 0.0001	1.4	< 0.0001	−3.1
Inpatient admission	1.6	< 0.0001	1.7	< 0.0001	1.9	< 0.0001	−4.3
Foregone inpatient care	0.8	< 0.0001	0.9	0.3622	0.8	0.0037	−3
**UEBMI: URBMI**							
Outpatient visit	1.4	< 0.0001	1.1	< 0.0001	1.0	0.0132	4.6
Inpatient admission	1.8	< 0.0001	1.6	< 0.0001	1.2	< 0.0001	1.6
Foregone inpatient care	0.8	0.0029	0.9	0.0011	0.8	< 0.0001	−3.1
**UEBMI: NRCMS**							
Outpatient visit	0.9	0.0004	1.0	0.4434	0.9	< 0.0001	1.4
Inpatient admission	1.3	< 0.0001	1.2	< 0.0001	1.0	0.0027	1.8
Foregone inpatient care	0.9	0.1070	0.9	< 0.0001	0.8	< 0.0001	3.2
**URBMI: NRCMS**							
Outpatient visit	0.7	< 0.0001	0.9	< 0.0001	0.9	< 0.0001	−3.2
Inpatient admission	0.7	< 0.0001	0.8	< 0.0001	0.9	< 0.0001	0.2
Foregone inpatient care	1.2	0.0169	1.0	0.6858	0.9	0.0024	6.3

^1^Ratios were generated by dividing the crude rates of healthcare utilisation between the comparison groups. The ratio indicates the gap between the comparison groups, where 1 implies no gap

^2^The difference in crude rates in 2008 minus the difference in crude rates in 2018 for the comparison groups

^3^*P*-values test for significant differences for each indicator in each year for the comparison groups

URBMI had a higher rate of outpatient visits than that of uninsured respondents in 2013 and 2018, and the gap in the rate of outpatient visits between URBMI and uninsured respondents increased between 2008 and 2018. URBMI had a higher rate of inpatient admission than that of uninsured respondents in 2008, 2013, and 2018, and the gap in the rate of inpatient admission between URBMI and uninsured respondents increased between 2008 and 2018. The rate of foregone inpatient care between URBMI and uninsured respondents was nearly at parity in 2008 and 2013, and as of 2018, URBMI had lower rates of foregone inpatient care than uninsured respondents did.

NRCMS had higher rates of outpatient visits and inpatient admissions and lower rates of foregone inpatient care than uninsured respondents did in 2008, 2013, and 2018. The gaps in the rates of outpatient visits and foregone inpatient care between NRCMS and uninsured respondents showed no statistically significant changes between 2008 and 2018, while the gap in the rate of inpatient admission increased between 2008 and 2018.

UEBMI had higher rates of outpatient visits and inpatient admissions than that of URBMI, while the gaps decreased between 2008 and 2018. As of 2018, the rates of outpatient visits were at parity between the UEBMI and URBMI. UEBMI had lower rates of foregone inpatient care than that of URBMI in 2008, 2013, and 2018, and the gaps were stable between 2008 and 2018.

Outpatient service utilisation was almost identical between UEBMI and NRCMS in 2008, 2013, and 2018. UEBMI had a higher rate of inpatient admission than NRCMS did in 2008 and 2013, while the inpatient admission rate reached parity in 2018, with no statistically significant difference detected between UEBMI and NRCMS. There was no statistically significant difference in the rates of foregone inpatient care between UEBMI and NRCMS in 2008, while the gaps in the rates of foregone inpatient care increased between 2013 and 2018. By 2018, UEBMI had a lower rate of foregone inpatient care than NRCMS did.

URBMI had lower rates of outpatient visits and inpatient admissions than NRCMS did in 2008, 2013, and 2018, while the gaps decreased between 2008 and 2018. URBMI had a higher rate of foregone inpatient care than NRCMS did in 2008. Rates of foregone inpatient care were nearly equal between URBMI and NRCMS in 2013, with a small but statistically significant difference in 2018.

### Changes in income-based disparities in healthcare utilisation within social health insurances

**Fig. 1:**
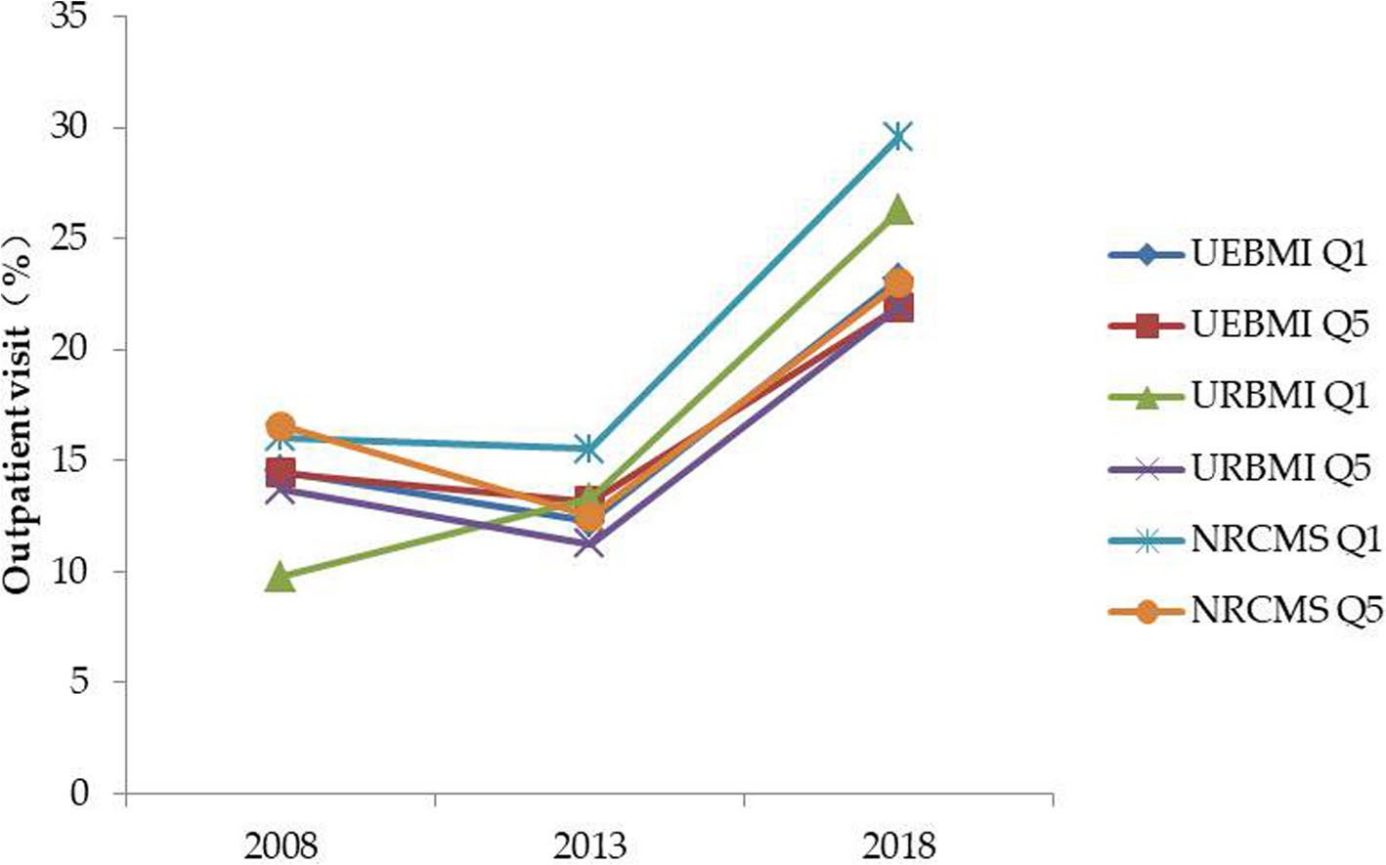
Trends in disparity in outpatient visit between Q1 and Q5. Q1: quintile 1 (poor); Q5: quintile 5 (least poor)

Figure 1 shows the absolute differences in outpatient visit between the poorest and best-off quintiles within UEBMI, URBMI, and NRCMS in 2008, 2013, and 2018. There were no statistically significant differences for outpatient visits between the poorest and wealthiest groups within UEBMI in 2008 and 2013, and were nearly equal in 2018, but the difference was statistically significant (see Table A4 in Additional file 1). The poorest groups within NRCMS and URBMI were more likely to use outpatient care than their wealthy counterparts in 2013 and 2018, despite a lack of differences (or even lower utilisation rate) in 2008 (Fig. 1).

**Fig. 2:**
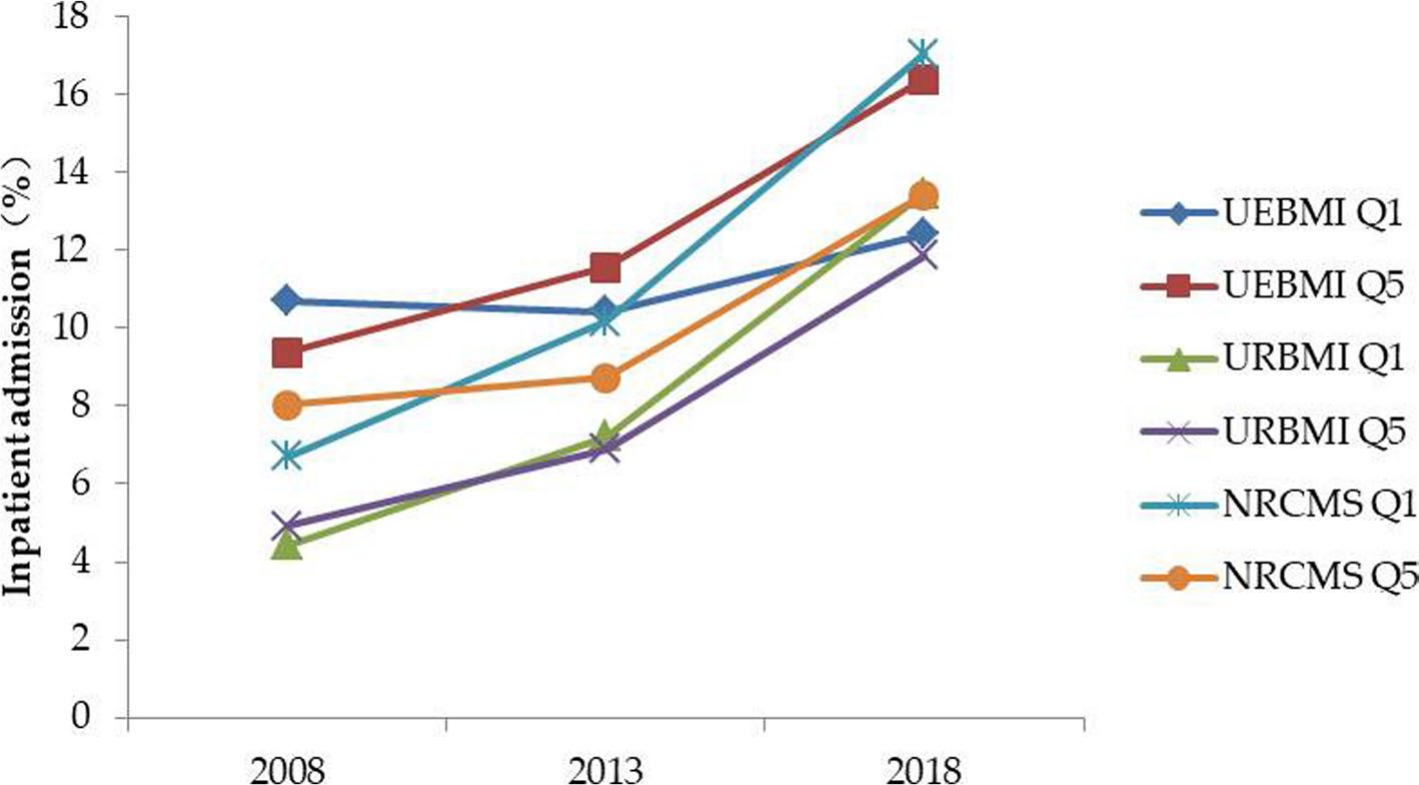
Trends in disparity in inpatient admission between Q1 and Q5. Q1: quintile 1 (poor); Q5: quintile 5 (least poor)

For inpatient admission, there were no statistically significant differences between the poorest and wealthiest groups within UEBMI in 2008 and 2013, while the poorest group was less likely to use inpatient care than the wealthiest group in 2018. The poorest groups within the URBMI and NRCMS were more likely to use inpatient care than their wealthy counterparts in 2018, despite a lack of differences (or even lower utilisation rate) in 2008. The gap in inpatient admission between the poorest group and wealthiest group within the NRCMS widened from 2013 to 2018 (Fig. 2).

**Fig. 3:**
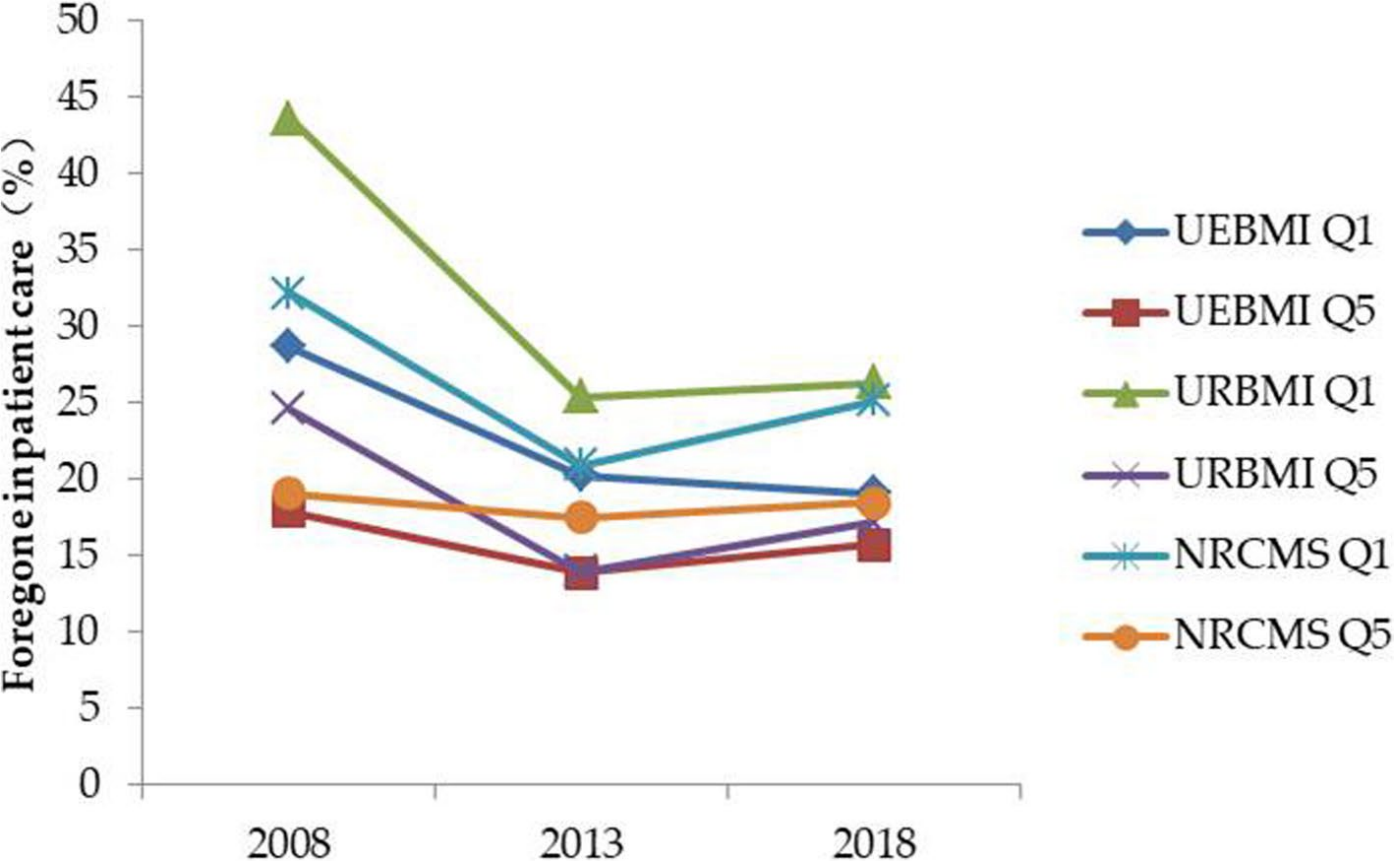
Trends in disparity in foregone inpatient care between Q1 and Q5. Q1: quintile 1 (poor); Q5: quintile 5 (least poor)

For the rate of foregone inpatient care, a large reduction occurred in the gap between the poor and wealthy in UEBMI, URBMI, and NRCMS (Fig. 3). The differences between the lowest and highest proportion of foregone inpatient care narrowed between 2008 and 2018, from a 25.7 to a 10.6 percentage point difference between the poorest in URBMI and the wealthiest in UEBMI (see Table A4 in Additional file 1). However, the poor groups in UEBMI, URBMI and NRCMS always experienced a higher rate of foregone inpatient care in comparison with their wealthy counterparts from 2008 to 2018.

## Discussion

This study analyses changes in disparities in healthcare utilisation between and within health insurances between 2008 and 2018. The results show that UEBMI, URBMI, and NRCMS had higher healthcare utilisation and experienced faster increases than uninsured respondents between 2008 and 2018. This implies that the rapid expansion of UEBMI, URBMI, and NRCMS with more generous benefits improved healthcare utilisation [2, 19, 26, 27]. This finding closely parallels existing empirical studies on URBMI and NRCMS [7, 28–30]. It is also in line with the international trend that health insurance plans in low- and middle-income countries generally promote healthcare utilisation [31]. Our results also show that gaps in outpatient visits and inpatient admissions narrowed across UEBMI, URBMI, and NRCMS from 2008 to 2018. This suggests improvements in insurance-related inequality in outpatient and inpatient utilisation. Huang et al. also found that disparities in healthcare utilisation between NRCMS and UEBMI decreased between 2010 and 2016 [6].

Our results also suggest improvements in income-based inequality in healthcare utilisation within URBMI and NRCMS, with faster increases in outpatient and inpatient utilisation and larger reductions in foregone inpatient care in the poorest groups than in the wealthiest ones. This finding is consistent with the theoretical analysis of moral hazard theory and previous empirical results. The moral hazard theory illustrates that health insurance plays a significant role in healthcare utilisation and distribution. The RAND experiment results showed that the price elasticity of healthcare service demand of low-income people is greater than that of high-income groups [32]. The change in healthcare service price caused by the compensation of health insurance has a greater impact on the demand and utilisation of healthcare services for low-income groups [33]. Existing empirical studies in China also showed that NRCMS and URBMI were associated with a reduction in inequalities in healthcare utilisation and underutilisation [14–17]. National data from CNHSS in 2008, 2013, and 2018 showed that respondents covered by URBMI and NRCMS with lower incomes were unhealthier than their high-income counterparts (see Tables A5 and A6 in Additional file 1), as is the result of data from Jiangsu Province [34]. People with more health service needs have more healthcare utilisation, suggesting equity in access to healthcare services [35]. With the re-classification of this study, the effects of the consolidation of the two schemes could also explained the remarkable increases in healthcare utilisation of URBMI and NRCMS, and the improvements in equalities in healthcare utilisation within them [7, 36]. For respondents covered by UEBMI, the wealthiest groups were unhealthier than the poorest group (see Tables A5 and A6 in Additional File 1). Our results show that the wealthiest group had a higher inpatient admission rate than the poorest group in 2018. However, we could not identify whether income-based inequity in inpatient admission improved within UEBMI because of the confounding factors of health service needs.

However, our results show gaps widened in foregone inpatient care between NRCMS and UEBMI from 2008 to 2018. The poorest groups (quintile 1) within URBMI and NRCMS were consistently more likely to forego admission to hospital recommended by doctors than their wealthy counterparts. The China Health and Retirement Longitudinal Study data also showed the lowest income group reported more foregone inpatient care than those in the highest income group [37]. It implies that although the poor have become more likely to use outpatient and inpatient care, they have always been more likely to abandon needed health services. The financial burden is still a serious consideration for the poor. National data from CNHSS in 2018 showed that the overall primary reason for foregoing inpatient admission was ‘Financial difficulties’ for respondents covered by URBMI and NRCMS, especially for the poorest groups within URBMI and NRCMS [38]. Empirical evidence has shown that the catastrophic health expenditure rate of those covered by NRCMS has increased significantly more than people covered by UEBMI and URBMI from 2000 to 2020 [39]. There are disparities in catastrophic health expenditure and impoverishment due to health expenditure among population income levels, and low-income households still have a higher chance of suffering financial hardship than high-income households [2].

It should be noted that the large increases in healthcare utilisation under the coverage of UEBMI, URBMI, and NRCMS are not synonymous with improvements in healthcare access because of inefficient utilisation. Moses et al.’s study indicated that China’s age-standardised outpatient utilisation (5.17 visits per capita) was close to the global average (5.42 visits per capita), but inpatient utilisation (0.14 admissions per capita) was significantly higher than the global average (0.10 admissions per capita) in 2016 [27]. Data from CNHSS in 2018 revealed that 4.5, 4.1, and 4.9% of inpatients under the coverage of UEBMI, URBMI, and NRCMS had gone through admission formalities immediately after discharge formalities but not leaving hospitals, which was significantly higher than that of uninsured inpatients (3.7%). Previous studies also reported inefficient utilisation behaviours, such as doctors induced decomposing hospitalisations, doctors and patients ‘conspiring’ with low standards of admission and treatment, ‘hanging bed’, and false cases [40, 41]. The National Healthcare Security Administration also exposed cases of cheating health insurance funds, such as bed-hanging hospitalisation, low-standard admission, and false medical cases [42].

This study has several limitations. First, although CNHSS sample was nationally representative, it could not be proved that the UEBMI, URBMI, NRCMS, and uninsured respondents within the surveys were representative of the target populations, as the characteristics of the UEBMI, URBMI, NRCMS, and uninsured population are absent from current public data. Second, the different months of the survey between 2008 and 2013 and 2018 might have led to the differences in health demand and healthcare utilisation of the respondents, which might also influence the disparities in healthcare utilisation between and within different health insurance groups. Third, this study did not incorporate all data but only two groups (the poorest and wealthiest groups) to investigate changes in income-based disparities in healthcare utilisation within social health insurances. Fourth, this study described the trends in insurance-related and income-related disparities in healthcare utilisation without controlling for other sociodemographic, economic, and health status variables. All of these are important issues to be addressed in future research.

## Conclusions

Remarkable increases in healthcare utilisation of URBMI and NRCMS, especially among the poorest groups, were accompanied by improvements in inequality in healthcare utilisation across UEBMI, URBMI, and NRCMS, and in income-based inequality in healthcare utilisation within URBMI and NRCMS. However, the poor groups in UEBMI, URBMI and NRCMS were consistently more likely to forego admission to hospital recommended by doctors from 2008 to 2018. The large increase in healthcare utilisation under the coverage of social health insurance might be accompanied by inefficient utilisation of health services. We suggest further focus on the foregoing admission care of the poor groups. China’s ongoing healthcare system reform should also focus on inefficient utilisation and quality of healthcare.

## Supplementary Information

**Additional file 1: Table A1.** Indicator Definition. **Table A2.** Trends in age-standardized healthcare utilisation by health insurances, 2008-2018. **Table A3.** Changes in absolute difference in healthcare utilisation between comparison groups, 2008-2018. **Table A4.** Changes in disparity (ratio) in healthcare utilisation between quintile 1 (Q1, poor) and quintile 5 (Q5, least poor) within the UEBMI, URBMI, and NRCMS: 2008, 2013, and 2018. **Table A5.** The prevalence of sick in the last 2 weeks of respondents covered by UEBMI, URBMI, and NRCMS: 2008, 2013, and 2018. **Table A6.** The prevalence of chronic diseases of respondents covered by UEBMI, URBMI, and NRCMS: 2013 and 2018.

**Additional file 2: Fig. A1.** Age-group compositions of UEBMI, URBMI, NRCMS, and uninsured respondents in 2008, 2013, and 2018.

## Data Availability

The datasets used during the current study are available from the corresponding author on reasonable request. All data analysed during this study are included in this published article and its supplementary information files.

## Abbreviations

UEBMI: Urban employee-based basic medical insurance
URBMI: Urban resident-based basic medical insurance
NRCMS: New rural cooperative medical scheme
URRBMI: Urban-Rural Resident Basic Medical Insurance
CNHSS: China National Health Services Survey
