# Supplementary material for: Trends in disparities in healthcare utilisation between and within health insurances in China between 2008 and 2018: a repeated cross-sectional study
Source: Int J Equity Health. 2022 Feb 24;21:30. doi: 10.1186/s12939-022-01633-4 (PMC8876177; doi:10.1186/s12939-022-01633-4)
Supplement: Supplementary file 1 — Additional file 1: Table A1. Indicator Definition. Table A2. Trends in age-standardized healthcare utilisation by health insurances, 2008-2018. Table A3. Changes in absolute difference in healthcare utilisation between comparison groups, 2008-2018. Table A4. Changes in disparity (ratio) in healthcare utilisation between quintile 1 (Q1, poor) and quintile 5 (Q5, least poor) within the UEBMI, URBMI, and NRCMS: 2008, 2013, and 2018. Table A5. The prevalence of sick in the last 2 weeks of respondents covered by UEBMI, URBMI, and NRCMS: 2008, 2013, and 2018. Table A6. The prevalence of chronic diseases of respondents covered by UEBMI, URBMI, and NRCMS: 2013 and 2018. [file 12939_2022_1633_MOESM1_ESM.docx]

Supplementary materials

Appendix A: Definitions of health insurance coverage statuses of respondents

In China, household registration and employment status determine residents’ social health insurance scheme [1, 2]. Eligible population for the urban employee-based basic medical insurance scheme (UEBMI) is urban employed residents, and Payroll taxes are the main funding source for UEBMI. Eligible population for urban resident-based basic medical insurance scheme (URBMI) is urban non-employed residents, including preschool children, students and other non-employed persons. Eligible population for the new rural cooperative medical scheme (NRCMS) is rural farmers, who may be employed or non-employed. Government subsidies and capitation premium are the major funding sources for URBMI and NRCMS. Participation policies stipulate that residents can participate in only one of the above social medical insurance schemes.

Integrating URBMI and NRCMS to Urban-Rural Resident Basic Medical Insurance (URRBMI), which was announced by central government in 2016 and gradually implemented nationwide, makes urban and rural non-employed residents participate in the same health insurance schemes regardless of household registration [1, 2]. Some areas piloted the integration of URBMI and NRCMS also with UEBMI (three- in-one) in terms of their administration organization but did not merge their fund pools with unified benefit packages. The existing integration policies reviewed showed that residents had more generous benefits with more comprehensive drug and service packages from the consolidation of the two schemes.

In the China National Health Services Survey (CNHSS), respondents were asked what kind of social health insurance schemes did they covered by. Taking into account the implementation of consolidating social health insurance schemes in China, the questionnaire of CNHSS in 2018 included five social health insurance coverage statuses: A. UEBMI; B. URBMI (not integrated with NRCMS); C. NRCMS (not integrated with URBMI); D. URRBMI (integrated at least at the management level); E. ‘three-in-one’ (the integration of UEBMI, URBMI, and NRCMS at least at the management level) [3]. For each of the above five social health insurance schemes, respondents were asked whether they were covered by it.

Taking into account the comparison of healthcare utilization of different social health insurance groups with the previous two surveys, the social health insurance coverage statue of respondents with URRBMI, ‘three-in-one’, or two or more of the five social health insurance schemes from CNHSS in 2018was redefined into UEBMI, URBMI, NRCMS, or other social health insurance scheme according to their household registration and occupation type. Detailed redefinition steps and rules are as follows:

1. If respondents’ answers to the coverage status of UEBMI were ‘yes’ (n=58490), then their social health insurance coverage status was defined as ‘UEBMI’, and their answers to the other four social health insurance schemes were assigned ‘no’.
2. If respondents’ answers to the coverage status of URBMI and NRCMS were both ‘yes’ (n=698) , then:
   1. if the respondents are ‘agricultural Hukou’ (n=480), then their social health insurance coverage status was defined as “NRCMS”, and their answers to the other four social health insurance schemes were assigned ‘no’;
   2. if the respondents are ‘non-agricultural Hukou’ (n=218), then their social health insurance coverage status was defined as ‘URBMI’, and their answers to the other four social health insurance schemes were assigned ‘no’;
3. If respondents’ answers to the coverage status of URBMI were ‘yes’ (n=27100), then their social health insurance coverage status was defined as ‘URBMI’, and their answers to the other four social health insurance schemes were assigned ‘no’.

4. If respondents’ answers to the coverage status of NRCMS were ‘yes’(n=65779), then their social health insurance coverage status was defined as ‘NRCMS’, and their answers to the other four social health insurance schemes were assigned ‘no’.

5. If respondents’ answers to the coverage status of URRBMI were ‘yes’ (n=91089), then:

- 1. if the respondents are ‘agricultural Hukou’ (n=57675), then their social health insurance coverage status was redefined as ‘NRCMS’, and their answers to the other four social health insurance schemes were assigned ‘no’;
  2. if the respondents are ‘non-agricultural Hukou’ (n=33352), then their social health insurance coverage status was redefined as ‘URBMI’, and their answers to the other four social health insurance schemes were assigned ‘no’;
  3. If the respondents’ household registration statuses were ‘unregistered’ or empty (n=62), then :

1. if the respondents were from rural area (n=43), then their social health insurance coverage status was redefined as ‘NRCMS’, and their answers to the other four social health insurance schemes were assigned ‘no’;
2. if the respondents were from urban area (n=19), then their social health insurance coverage status was redefined as ‘URBMI’, and their answers to the other four social health insurance schemes were assigned ‘no’.

6. If respondents’ answers to the coverage status of ‘three-in-one’ were ‘yes’ (n=5041), then:

- 1. If the respondents are military personnel in active service (n=2), then their social health insurance coverage status was redefined as other social health insurance.
  2. If the respondents are civil servants (n=31), professional and technical personnel (n=302), staff (n=581), enterprise management personnel (n=172), or workers (n=600), then their social health insurance coverage status was redefined as ‘UEBMI’;
  3. If the respondents are farmers (n=354), then their social health insurance coverage status was redefined as ‘NRCMS’;
  4. If the respondents are freelancers (n=414), self-employed (n=303), or others (n=307), or their employment statuses were empty (n=1975), then:

1. If the respondents are ‘agricultural Hukou’ (n=1090), then their social health insurance coverage status was redefined as ‘NRCMS’;
2. If the respondents are ‘non-agricultural Hukou’ (n=1909), then their social health insurance coverage status was redefined as ‘URBMI’.

Appendix B

Table A1. Indicator Definition

| **Indicator** | **Definition** |
| --- | --- |
| Outpatient visit | Rate of household members who sought outpatient care at any facility during the two weeks before the survey. |
| Inpatient admission | Rate of household members admitted to a hospital in the year before the survey. |
| Foregone inpatient care | Percent of household members reported having refused to be admitted to hospital against medical advice in the year before the survey. |

Appendix C

Table A2. Trends in age-standardized healthcare utilisation by health insurances, 2008-2018

|  | **2008** | **2013** | **2018** | **2008-13** | **2013-18** | **2008-18** |
| --- | --- | --- | --- | --- | --- | --- |
| **Outpatient visit** | 9.4 | 9.0 | 15.2 | -0.4 | 6.2 | 5.8 |
| UEBMI | 10.6 | 11.1 | 19.4 | 0.5 | 8.3 | 8.8 |
| URBMI | 14.7 | 11.3 | 20.2 | -3.4 | 8.9 | 5.5 |
| NRCMS | 10.2 | 8.1 | 15.3 | -2.0 | 7.2 | 5.2 |
| Uninsured | 9.4 | 9.0 | 15.2 | -0.4 | 6.2 | 5.8 |
| **Inpatient admission** |  |  |  |  |  |  |
| UEBMI | 5.5 | 7.1 | 10.2 | 1.7 | 3.0 | 4.7 |
| URBMI | 5.2 | 6.6 | 10.8 | 1.3 | 4.3 | 5.6 |
| NRCMS | 6.9 | 8.2 | 12.0 | 1.3 | 3.7 | 5.1 |
| Uninsured | 4.3 | 5.0 | 6.9 | 0.7 | 1.8 | 2.5 |
| **Foregone inpatient care** |  |  |  |  |  |  |
| UEBMI | 13.8 | 12.5 | 14.6 | -1.3 | 2.0 | 0.8 |
| URBMI | 22.8 | 17.2 | 16.9 | -5.6 | -0.3 | -5.9 |
| NRCMS | 20.1 | 14.9 | 18.0 | -5.2 | 3.1 | -2.1 |
| Uninsured | 28.6 | 22.7 | 27.8 | -5.9 | 5.0 | -0.8 |

Note: We used direct method to calculate age-standardized utilization rates. The 2010 census population of China was used as the standard population. Age-standardized utilization rates can be calculated for (I) age groups:

ASR=$\frac{\sum_{i=1}^{I} (r_{i}\times w_{i})}{\sum_{i=1}^{I} w_{i}}$, where I is the 8 age groups ( i=1 to 8) : <5, 5-14, 15-24, 25-34, 35-44, 45-54, 55-64, ≥65; $r_{i}$ is the age-specific utilization rate; $w_{i}$ is the age-specific standard population.

**Appendix D**

**Table A3.** Changes in absolute difference in healthcare utilisation between comparison groups, 2008-2018

| **Comparison groups** | **2008** | **2013** | **2018** |
| --- | --- | --- | --- |
| **UEBMI : Uninsured** |  |  |  |
| Outpatient visit | 3.8 | 4.5 | 5.5 |
| Inpatient admission | 4.9 | 5.9 | 7.4 |
| Foregone inpatient care | -9.4 | -3.6 | -9.6 |
| **URBMI : Uninsured** |  |  |  |
| Outpatient visit | -0.3 | 3.5 | 6.0 |
| Inpatient admission | 0.8 | 1.8 | 4.9 |
| Foregone inpatient care | -3.1 | -1.0 | -6.4 |
| **NRCMS : Uninsured** |  |  |  |
| Outpatient visit | 4.7 | 4.4 | 7.8 |
| Inpatient admission | 2.6 | 3.7 | 6.9 |
| Foregone inpatient care | -7.9 | -1.3 | -4.9 |
| **UEBMI : URBMI** |  |  |  |
| Outpatient visit | 4.1 | 1.0 | -0.5 |
| Inpatient admission | 4.1 | 4.1 | 2.5 |
| Foregone inpatient care | -6.3 | -2.6 | -3.2 |
| **UEBMI : NRCMS** |  |  |  |
| Outpatient visit | -0.9 | 0.1 | -2.3 |
| Inpatient admission | 2.3 | 2.2 | 0.5 |
| Foregone inpatient care | -1.5 | -2.3 | -4.7 |
| **URBMI : NRCMS** |  |  |  |
| Outpatient visit | -5.0 | -0.9 | -1.8 |
| Inpatient admission | -1.8 | -1.9 | -2.0 |
| Foregone inpatient care | 4.8 | 0.3 | -1.5 |

**Appendix E**

**Table A4.** Changes in disparity (ratio) in healthcare utilisation between quintile 1 (Q1, poor) and quintile 5 (Q5, least poor) within the UEBMI, URBMI, and NRCMS: 2008, 2013, and 2018.

|  | **2008** | **p value** | **2013** | **p value** | **2018** | **p value** |
| --- | --- | --- | --- | --- | --- | --- |
| Outpatient visit |  |  |  |  |  |  |
| UEBMI | 1.0 | 0.8987 | 0.9 | 0.0553 | 1.1 | 0.0296 |
| URBMI | 0.7 | 0.0018 | 1.2 | 0.0002 | 1.2 | <0.0001 |
| NRCMS | 1.0 | 0.1112 | 1.2 | <0.0001 | 1.3 | <0.0001 |
| Percentage point gap between lowest and highest frequency | 6.8 |  | 4.3 |  | 7.7 |  |
| Inpatient Admission |  |  |  |  |  |  |
| UEBMI | 1.1 | 0.0623 | 0.9 | 0.0129 | 0.8 | <0.0001 |
| URBMI | 0.9 | 0.558 | 1.1 | 0.4147 | 1.1 | 0.0002 |
| NRCMS | 0.8 | <0.0001 | 1.2 | <0.0001 | 1.3 | <0.0001 |
| Percentage point gap between lowest and highest frequency | 6.3 |  | 4.7 |  | 5.2 |  |
| Foregone inpatient care |  |  |  |  |  |  |
| UEBMI | 1.6 | <0.0001 | 1.5 | <0.0001 | 1.2 | 0.0097 |
| URBMI | 1.8 | 0.0091 | 1.8 | <0.0001 | 1.5 | <0.0001 |
| NRCMS | 1.7 | <0.0001 | 1.2 | 0.0006 | 1.4 | <0.0001 |
| Percentage point gap between lowest and highest frequency | 25.7 |  | 11.5 |  | 10.6 |  |

**Appendix F**

**Table A5.** The prevalence of sick in the last two weeks of respondents covered by UEBMI, URBMI, and NRCMS: 2008, 2013, and 2018.

| **Quintile** | **UEBMI** | | | **URBMI** | | | **NRCMS** | | |
| --- | --- | --- | --- | --- | --- | --- | --- | --- | --- |
|  | **2008** | **2013** | **2018** | **2008** | **2013** | **2018** | **2008** | **2013** | **2018** |
| Poorest | 23.7 | 26.3 | 31.3 | 15.2 | 21.1 | 33.7 | 18.4 | 20.4 | 37.4 |
| 2nd | 24.5 | 26.9 | 32.8 | 11.5 | 19.1 | 29.6 | 16.9 | 16.7 | 32.0 |
| 3nd | 25.2 | 29.6 | 35.7 | 12.4 | 18.8 | 29.9 | 16.2 | 15.1 | 30.3 |
| 4nd | 23.5 | 30.4 | 37.7 | 13.2 | 18.6 | 28.9 | 16.6 | 15.9 | 29.8 |
| Richest | 21.9 | 29.8 | 37.1 | 14.8 | 16.2 | 28.4 | 17.7 | 16.0 | 30.2 |

**Table A6.** The prevalence of chronic diseases of respondents covered by UEBMI, URBMI, and NRCMS: 2013 and 2018.

| **Quintile** | **UEBMI** | | | **URBMI** | | | **NRCMS** | | |
| --- | --- | --- | --- | --- | --- | --- | --- | --- | --- |
|  | **2013** | **2018** | **2013-18** | **2013** | **2018** | **2013-18** | **2013** | **2018** | **2013-18** |
| Poorest | 28.3 | 30.5 | 2.2 | 27.5 | 38.2 | 10.7 | 28.5 | 42.7 | 14.2 |
| 2nd | 29.1 | 32.0 | 2.9 | 23.8 | 32.4 | 8.6 | 22.0 | 35.4 | 13.3 |
| 3nd | 32.1 | 35.5 | 3.4 | 24.0 | 32.0 | 8.1 | 19.9 | 32.7 | 12.7 |
| 4nd | 32.8 | 37.1 | 4.3 | 23.5 | 31.7 | 8.2 | 19.9 | 31.2 | 11.3 |
| Richest | 31.9 | 36.9 | 5.0 | 20.5 | 30.4 | 9.9 | 19.9 | 30.8 | 10.9 |

References

1. Meng, Q.Y., et al., *Consolidating the social health insurance schemes in China: towards an equitable and efficient health system.* Lancet, 2015. **386**(10002): p. 1484-1492.

2. Wang, Z., Chen, Y., Pan, T. , Liu, X., Hu, H., *The comparison of healthcare utilization inequity between URRBMI and NCMS in rural China.* Int J Equity Health 2019. **18**(90).

3. Center for Health Statistics and Information NHC, *An Analysis Report of National Health Service Survey in China, 2018*. 2021, Beijing,China: People's medical publishing house.
